# Ultrasound imaging of core muscles activity in multiparous women with vaginal laxity: a cross-sectional study

**DOI:** 10.1038/s41598-024-58955-2

**Published:** 2024-04-20

**Authors:** Doaa A. Abdel Hady, Omar M. Mabrouk, Doaa A. Osman

**Affiliations:** 1 Department of Physical Therapy for Women’s Health, Faculty of Physical Therapy, Deraya University, Minia, Egypt; 2 Department of Basic Science, Faculty of Physical Therapy, Deraya University, Minia, Egypt; 3 Department of Physical Therapy for Women’s Health, Faculty of Physical Therapy, Cairo University, Giza, Egypt

**Keywords:** Ultrasound imaging, Core muscles, Multiparous women, Vaginal laxity, Health care, Health occupations

## Abstract

Vaginal laxity (VL) is a common condition among multiparous women, especially those who have delivered vaginally. Since pelvic floor muscles (PFMs) work synergistically with other core muscles, physical therapy protocols that aim to treat VL should train the PFMs in combination with other core muscles. To investigate the activity of core muscles in multiparous women with and without VL, and its relation to sexual function. An observational, cross-sectional study. The study included 100 multiparous women, who were divided into two groups according to their scores on the vaginal laxity questionnaire (VLQ). Women who scored between 1 and 3 on the VLQ were categorized as having VL (n = 48), while those who scored between 5 and 7 were placed in the control group (n = 52). The primary outcomes were PFM displacement, diaphragmatic excursion, transversus abdominis activation ratio, and lumbar multifidus thickness measured by ultrasound imaging. The secondary outcome was sexual functioning, evaluated using the Arabic female sexual function index (ArFSFI). The VL group had significantly lower PFM displacement (mean difference (MD) − 0.42; 95% confidence interval (CI) − 0.49 to − 0.33; p = 0.001), diaphragmatic excursion (MD − 2.75; 95% CI − 2.95 to − 2.55; p = 0.001), lumbar multifidus thickness (MD − 10.08; 95% CI − 14.32 to − 5.82; p = 0.02), and ArFSFI scores (MD − 9.2; 95% CI − 10.59 to − 7.81; p = 0.001) in comparison to the control group (p < 0.05). Nevertheless, the transversus abdominis activation ratio demonstrated no significant difference between the two groups (MD 0.06; 95% CI − 0.05 to 0.17; p = 0.33). Multiparous women with VL had significantly lower PFM displacement, diaphragmatic excursion, lumbar multifidus thickness, and sexual function index scores than women in the control group. The only exception was transversus abdominis activation, which did not differ significantly between the VL and control groups.

## Introduction

Vaginal laxity (VL) is characterized by the International Continence Society and the International Urogynecological Association as an excessive looseness of the vaginal tissue^1^. It adversely impacts women’s sexual functioning, urinary continence, and overall quality of life^2^. Research findings indicate that a considerable percentage (28–40%) of females receiving care at clinics specializing in urogynecology experience laxity in the vaginal area to an extent that impacts their overall well-being^3,4^.

Pregnancy and vaginal delivery are linked to VL; however, its exact cause is not known. Throughout pregnancy, the uterus grows and both uterine and fetal weights significantly increase. This results in added stress on the pelvic muscles, leading to their relaxation and weakening. Hormonal changes, such as elevated levels of relaxin, along with the degradation of elastin in the tissues of pelvic floor also contribute to the relaxation of pelvic floor^5^. Moreover, the vaginal childbirth procedure, involving vaginal wall stretching and trauma to the pelvic floor muscles (PFMs), can further contribute to the development of VL^6^.

The pelvic floor involves a group of muscles and connective tissues in a dome-shaped structure that encloses the anal, vaginal, and urethral pathways. Proper PFM contraction and relaxation are essential for supporting internal organs and are essential for functions like bowel and bladder control, bowel movements, micturition, sex, and vaginal birth^7^. PFM contraction has been linked to increased orgasmic and sexual reactions. Females who have PFM weakness and undergo retraining for muscle strengthening have reported improved sexual experiences^8,9^. PFM strengthening could be a helpful physiotherapy strategy for managing VL^9,10^.

The PFMs are among the primary core muscles, alongside the diaphragmatic, transversus abdominis, and lumbar multifidus muscles. The interplay of the aforementioned muscles forms a cylindrical structure that collaborates to generate forces within the lumbopelvic region, leading to enhanced stability and control of the trunk, fostered movement effectiveness, better balance, harmonized coordination, heightened postural alignment and stability, as well as maintenance of continence^11,12^. Despite this synergistic action between the PFMs and other core muscles, current rehabilitation programs for VL focus on PFM training^9,10^. No previous studies have evaluated the core muscle activity in women with VL to determine which additional core muscle groups should be targeted for training beyond just the PFMs.

Ultrasound imaging has achieved tremendous popularity in both research and clinical contexts, especially within the field of physical therapy, owing to its noninvasive capability of quantifying muscle shape and response^13^. It utilizes an innovative methodology to assess the morphological characteristics of deep core muscles,including the PFMs^14^, diaphragm^15^, transversus abdominis^16^, and lumbar multifidus^17^. By visually examining the deep muscles’ location and shape during the measurement process, ultrasound imaging ensures the acquisition of accurate and highly reliable data^16^. Consequently, this study utilized ultrasound imaging to evaluate the activity of the core musculature, including the PFMs, diaphragm, transversus abdominis, and lumbar multifidus, in multiparous women with and without VL. Another aim was to compare the sexual functioning between the vaginal laxity and control groups. It was hypothesized that there would be a difference in the core muscle activity between multiparous women with VL and those without VL. Specifically, we hypothesized that women with VL would have weaker core muscle activity compared to women without VL.

## Subjects and methods

### Design

This study employed an observational cross-sectional design and received ethical approval from the institutional review board of the Faculty of Physical Therapy, Cairo University [No: P.T.REC/012/004230], and was given a ClinicalTrials.gov ID NCT05707793. It was conducted following the Helsinki Declaration’s criteria for human research.

### Recruitment

A total of 100 women were selected to take part in this study. Recruitment of women experiencing VL was via referrals from Minia University Hospital’s gynecological outpatient clinic, in Minia, Egypt. Women in the control group were enrolled from Minia’s Family Planning clinics, in Egypt. Participants evaluated themselves by the Vaginal Laxity Questionnaire (VLQ). Individuals who scored between 1 and 3 on the VLQ in addition to reporting symptoms such as vaginal gas, entrapped water, reduced friction during intercourse, and labia majora laxity were categorized into the VL group^18,19^, while those who scored between 5 and 7 on the VLQ were placed in the control group^19^.

### Participant selection criteria

Participants had to meet certain requirements to be considered eligible for the study, which included being married women, having coitus at least once monthly^20^, maintaining an inactive lifestyle defined as less than 2.5 h/week of moderate physical activity^21^, having an age range of 35–45 years^22^, and maintaining a body mass index (BMI) between 25 and 30 kg/m^2^. Additionally, participants were required to have delivered babies vaginally twice to three times, as VL is commonly associated with a higher parity number and vaginal childbirth^23^. Moreover, they were required to have a minimum 2-year gap since their last delivery. Individuals with urinary incontinence, genital organ descent, vesicovaginal or rectovaginal fistulation, dysfunctional uterine bleeding, cystitis, urethritis, confirmed positive pregnancy result, previously assisted childbirth (ventouse or forceps), herniated spinal disc, issues with pelvic area or lower extremities, unequal leg lengths, abdominal muscle diastasis, and past surgical procedures involving spine, abdomen, or pelvis, as well as diabetic, heart-related, or respiratory disorders, and those using a uterine device for contraception, or consuming medications for sexual health or medications affecting tissue regeneration (like chemotherapeutic agents, psychiatric drugs, corticosteroids, and anti-inflammatory medications) were not allowed to participate in the study.

### Group categorization

**Figure 1:**
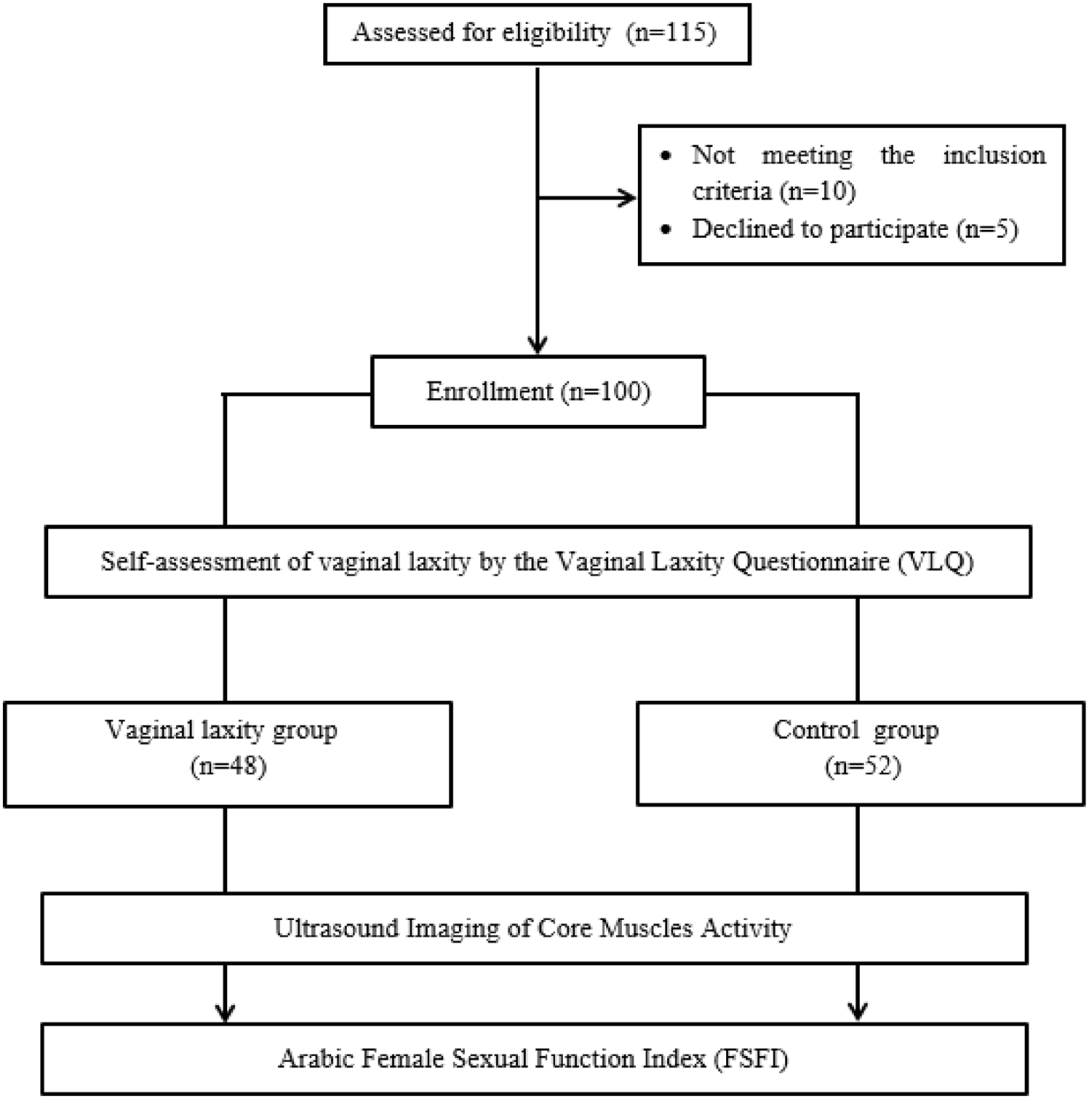
The study’s flow chart.

Participants were categorized into two groups relying on their scoring on the VLQ, the sole tool that exists for evaluating VL^24^. They were requested to assess their VL via a rating scale that spanned from 1 to 7, with 1 indicating “very loose”, 2 indicating “moderately loose”, 3 indicating “slightly loose”, 4 indicating “neither loose nor tight”, 5 indicating “slightly tight”, 6 indicating “moderately tight”, and 7 indicating “very tight”. The VL group comprised 48 women who reported scores between 1 and 3, reflecting the presence of VL. Meanwhile, the control group comprised 52 participants who scored between 5 and 7, signifying the absence of VL^19^. Figure 1 provides an illustration of the study’s flow chart. After the details of the study were described, including its purpose and potential benefits, every participant signed a consent form, safeguarding the confidentiality of their data and granting them the freedom to retract their approval at any point.

### Outcome measures

#### Primary outcome measures

##### Ultrasound imaging of core muscles activity

A skilled physical therapist with 5 years of expertise in ultrasound-based diagnostic and therapeutic imaging, along with a certified postgraduate diploma in ultrasound methodologies, conducted all ultrasonographic measurements. Ultrasonographic imaging equipment (Mindray DP10, Curvilinear probe, with a frequency of 2–5 MHz, Serial number: bn-75013216, China) was employed to assess PFM displacement, diaphragmatic excursion, transversus abdominis activation ratio, and lumbar multifidus thickness. Three measurements were averaged for data analysis. The measurement procedure was conducted following 5 min of presentation in a relaxed supine position. Each participant received instructions about how to correctly apply contraction of all examined muscles to create familiarization during the assessment^25^.

**Figure 2:**
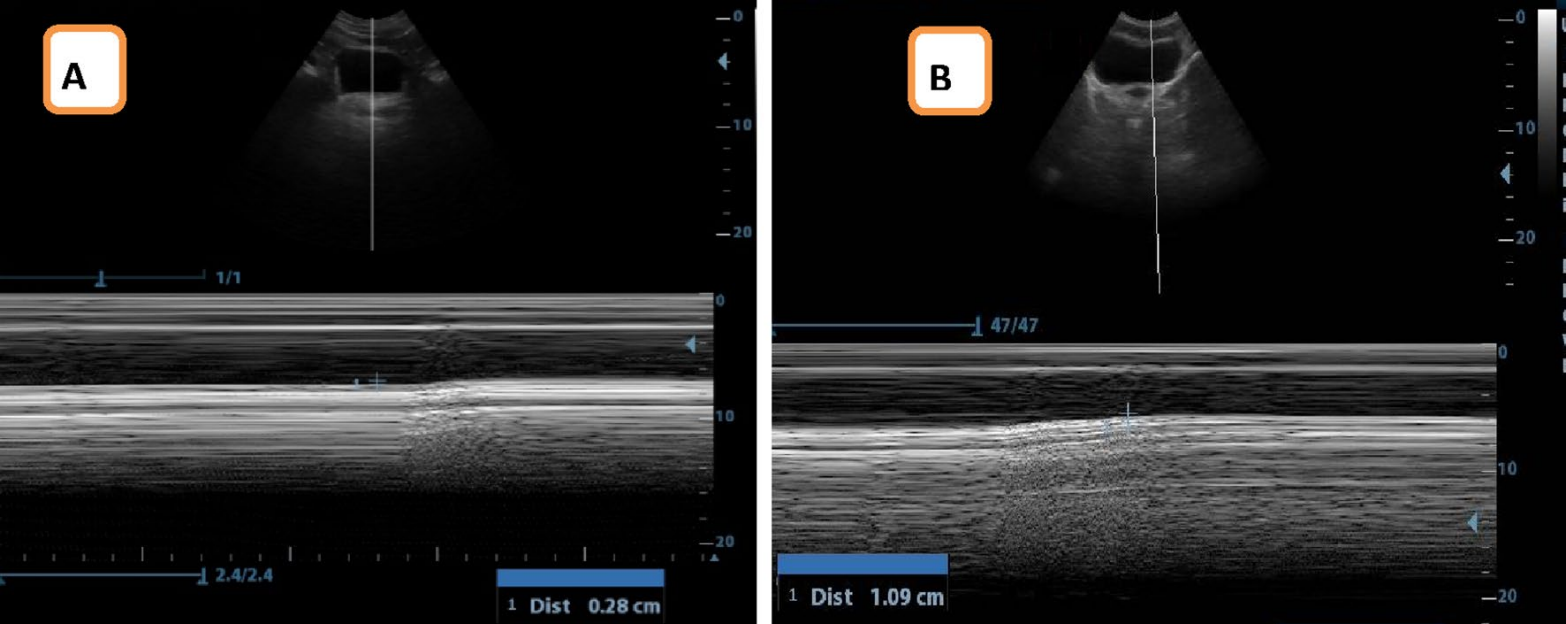
Ultrasound measurement of PFM displacement (**A**) VL group, (**B**) control group.

*Assessment of PFM displacement* For evaluating PFM displacement in the two groups, an ultrasound imaging unit incorporating a 5 MHz curvilinear probe was utilized. Measurements followed a consistent bladder-filling protocol, where participants were directed to evacuate their bladders before the test by an hour. Subsequently, they drank 450 to 500 ml of water and refrained from urination until after the evaluation. This ensured a clear acoustic window for optimal results. Participants assumed a crock-lying position with their lower back in a neutral alignment while their hips and knees were in 60° flexion. The transducer was positioned in a horizontal direction over the pubic symphysis, directly at the abdominal center, with an angle of approximately 60° from the vertical^14^. Transabdominal ultrasound relies on evaluating the bladder base movement as a marker for PFM activity. This technique enables the assessment of the quality of PFM contractions. Its practicality lies in its non-invasive nature, as it does not necessitate exposure of intimate body parts, making it suitable for clinical use across genders and age groups due to its speed and ease of application^26^. For evaluating the displacement of the posterior bladder wall resulting from PFM contractions, participants were directed to execute three maximal PFM contractions. PFM ultrasound evaluations were conducted using motion mode (M-mode). In the transverse plane, the inferior border of the bladder was utilized as a reference point (indicated by the anechoic margin end and the hyperechoic line beginning representing the pelvic floor deep plane) and then instruct the participant to maximally contract the PFM by squeezing around the urethra, anus, and vagina as if preventing loss of urine and flatulence, without contracting abdominal or leg muscles, followed by screen freeze and use calipers to measure the vertical distance of bladder base displacement which represented by hyperechoic line (Fig. 2). To maintain a consistent visual field during both rest and maximal contraction, the transducer remained in a steady position during the entire procedure^27^. Transabdominal ultrasound represents a valid and reliable method for assessing the PFMs (ICC_0.84)^28^.

**Figure 3:**
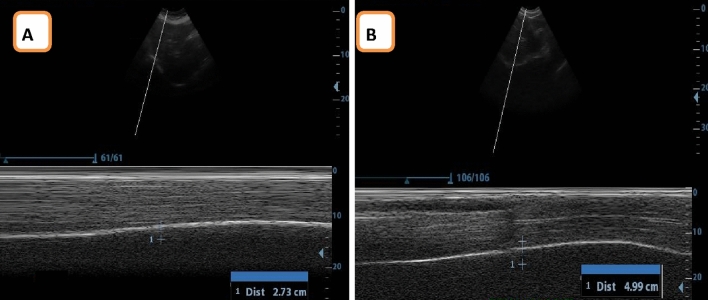
Ultrasound measurement of Diaphragmatic excursion (**A**) VL group, (**B**) control group.

*Assessment of diaphragmatic excursion* M-mode ultrasound imaging 2.5–5 MHZ curvilinear probe was utilized to evaluate the diaphragmatic excursion of the right hemi diaphragm for each participant in the two groups while assuming a supine position. The transducer was placed between the mid-clavicular line and the anterior axillary line, just below the right costal edge. It was then directed in medial, cephalic, and dorsal ways to capture the posterior portion of the hemidiaphragm on the right side. Measurements of the diaphragmatic excursion were obtained by positioning calipers at the top and bottom of the diaphragmatic inspiratory slope. All measurements were recorded at the expiratory phase end^15^ (Fig. 3).

Diaphragmatic excursion measured using ultrasound provides excellent temporal resolution, reproducibility, and accuracy. The intra-observer agreement for diaphragmatic excursion showed high consistency, with ICC falling within the range of 0.876 to 0.999. Similarly, the inter-observer agreement demonstrated a strong level of agreement, with ICC ranging from 0.76 to 0.989, as reported in reference^29^.

**Figure 4:**
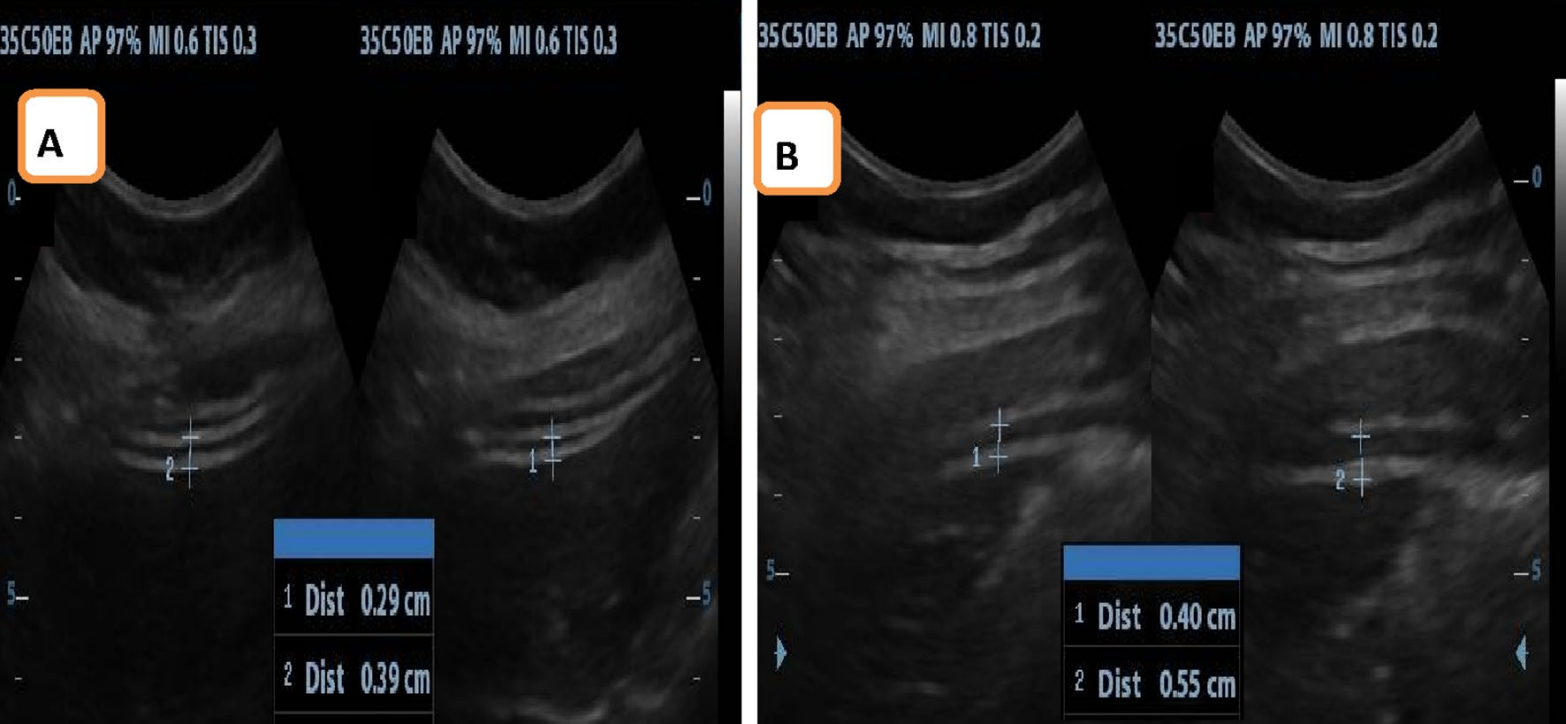
Ultrasound measurement of transversus abdominis activation ratio (**A**) VL group, (**B**) control group.

*Assessment of transversus abdominis activation ratio* For evaluating the transversus abdominis activation ratio of the left side for all participants in both groups, ultrasound images were captured using a 5 MHz curvilinear transducer from a supine-lying position. The transducer was positioned at the middle point between the lower ribcage and the anterior superior iliac spine along the anterior axillary line in a transverse plane. The measurement of transversus abdominis activation ratio was determined by recording the distance between calibers at the lower border of the internal obliques muscle and the other on the lower hyperechoic line of the peritoneum during rest, then applying splitting screen mode to measure thickness during abdominal drawing in maneuvers (ADIM) for all participants. The ratio of activation was obtained through the division of the thickness during ADIM by the thickness at rest^30^ (Fig. 4). The average of three measurements was taken. The intra-image reliability for transversus abdominis contraction thickness and ratio was excellent, with ICCs ranging from 0.95 to 1.00. However, the inter-image reliability for absolute muscle thickness was slightly lower, with ICCs ranging from 0.77 to 0.97^31^.

**Figure 5:**
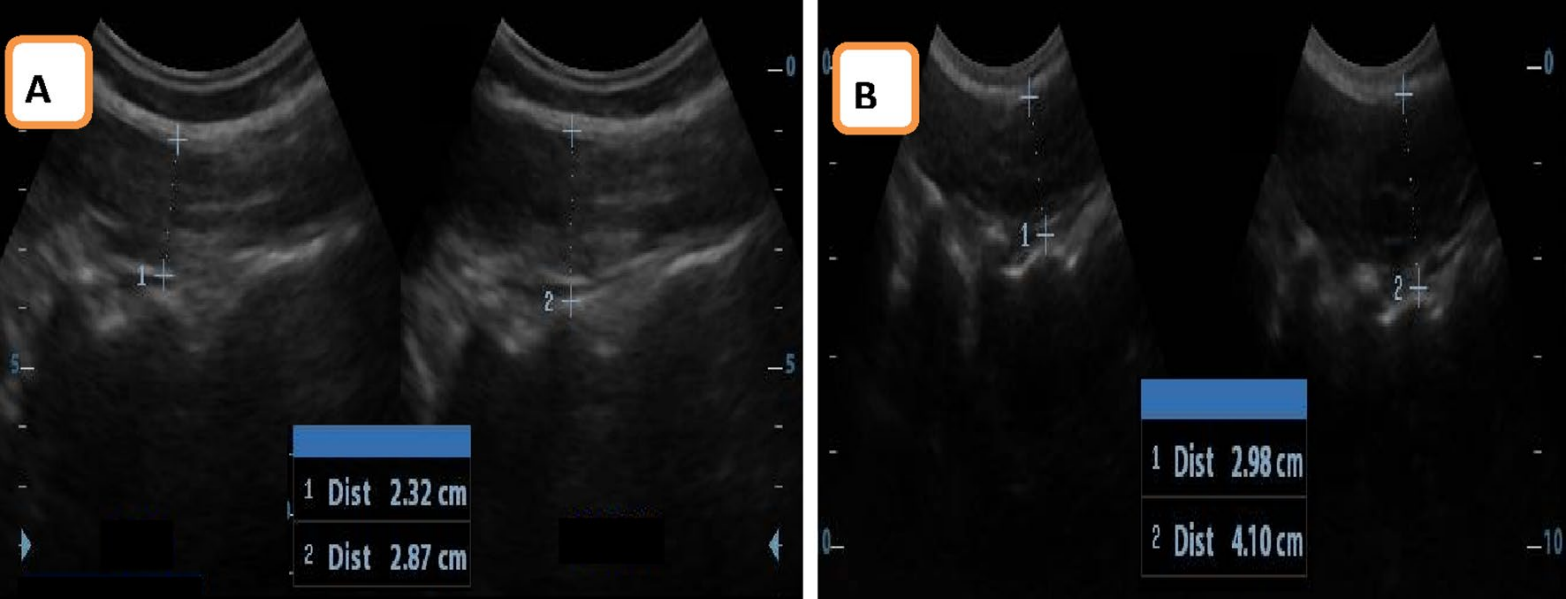
Ultrasound measurement of lumbar multifidus (**A**) VL group, (**B**) control group.

*Assessment of lumbar multifidus thickness* Ultrasound imaging 3–7 MHZ curvilinear transducer was used to evaluate the left lumbar multifidus thickness for each participant in both groups. The measurement of lumbar multifidus thickness at the level of L4–L5 was conducted in both resting and contracted positions^17,32^. In the resting position, the measurement was taken while the participant lay face down, utilizing a pillow below the abdominal region to reduce lumbar lordosis. During the contracted position, the measurement was captured as the participant executed the contralateral arm lift task from a prone-lying position. For this task, the participant was instructed to raise her right upper body about 5 cm from the table while repositioning her upper extremities overhead, with elbow flexion to 90° and shoulder abduction to 120°, as assessed by a goniometer. To determine the lumbar multifidus level, the transducer was positioned lengthwise along the lumbar spine, with its center positioned over the relevant spinous processes. By distinguishing the sacrum as a vertically oriented structure and contrasting it with the shorter and curved spinous processes, the desired level was identified. Subsequently, the transducer was adjusted laterally and slightly angled inwards until the targeted facet joint became visible. During this step, the transducer was directly above the lumbar multifidus, and measurement was captured from the facet joint apex to the plane situated between the thoracolumbar fascia and the subcutaneous fat^17^ (Fig. 5). The measurement of lumbar multifidus thickness was reported as a percent of change or ratio between rest and contraction thickness, which serves as an indicator of muscle activation. It was calculated utilizing the following equation^33^:

$$\text{Lumbar multifidus thickness}=\left(\frac{\text{ Thickness at contraction}-\text{Thickness at rest}}{\text{Thickness at rest}}\right).$$

#### Secondary outcome measure

##### Arabic female sexual function index (ArFSFI)

The ArFSFI is a concise and comprehensive self-report tool consisting of 19 items. It was used to evaluate the six essential elements of female sexual functioning in both groups, including desire, arousal, lubrication, orgasm, satisfaction, and pain. The index offered different response choices: the initial two questions had five possible options, while other questions were evaluated using a scale ranging from zero to five. Scores were then calculated by combining the responses and multiplying them by specific factors (0.6 for the first two items, 0.3 for items 3–10, and 0.4 for items 11–19). The total score ranged between 2 and 36, with scores exceeding 28.1 indicating satisfactory sexual health and scores lower than 28.1 suggesting compromised sexual functioning^34^.

The ArFSFI has been validated, shown reliability, and gained local acceptance as an assessment method for sexual dysfunction within the Egyptian female population. The reliability of test-retests demonstrated high consistency in the total score and various domain scores, with values in the range of 0.92 to 0.98. The domains also exhibited strong internal consistency, with values in the range of 0.85 to 0.94. Moreover, this tool exhibited high divergent validity. In terms of overall performance, this tool achieved an excellent result, achieving an area under the curve of 0.985 with a 95% confidence interval ranging from 0.978 to 0.992^35^.

##### Calculation of sample size and data analysis

A sample size determination was conducted based on the results of PFM displacement as reported from a pilot study. It was calculated by G*Power^36^, according to the statistical indices d = 0.5, with an effect size dz equal to 0.5, (1 − B) = 0.95 power analysis, and two side 5% significant level. Hence, the minimum estimated sample size encompassed 90 women, and it was increased to 100 women to exclude subject dropout. The Statistical Package for the Social Sciences (SPSS) version 25 for Windows was utilized to conduct all statistical analyses. Results are displayed as either the mean along with the standard deviation or as the median within a range of values (minimum to maximum). To analyze the differences in participant characteristics between the groups, an unpaired t-test was applied. The assessment of data normality was conducted utilizing the Shapiro–Wilk test. Additionally, Levene’s test was employed to examine the homogeneity of variances between the groups. To compare the PFM displacement, diaphragmatic excursion, transversus abdominis activation ratio, lumbar multifidus thickness, and ArFSFI between the two groups, unpaired t-tests were utilized. Meanwhile, the VLQ was compared using the Mann–Whitney test. A significance level of p < 0.05 was established for all conducted statistical tests.

### Ethical statement

All relevant international, national, and institutional guidelines for the ethical treatment of human participants are considered. Approval adheres to the principles outlined in the Declaration of the Council of the International Organization for Medical Science, the guidelines of the World Health Organization, and the Egyptian Clinical Trial Law from April 2018.

### Consent statement

All participants included in this study provided their informed consent.

## Results

### Participant characteristics

Age, BMI, and parity showed no statistically significant distinctions between both groups (p > 0.05). Nevertheless, there was a notable statistical difference in VLQ scores (p < 0.05) (Table 1).

**Table 1:** Participant characteristics of both groups.

	VL group (n = 48)	Control group (n = 52)	p-value
Age (years)	40.26 ± 3.27	39.48 ± 3.39	0.24
BMI (Kg/m^2^)	27.22 ± 2.07	26.85 ± 2.14	0.38
Parity	2.46 ± 0.50	2.35 ± 0.48	0.26
VLQ	2 (1–2)	5 (4–6)	0.01*

Values are expressed as mean ± standard deviation for age, BMI, and parity, and as median (minimum–maximum) for VLQ.

*BMI* body mass index, *VLQ* vaginal laxity questionnaire, *VL* vaginal laxity, *p* probability.

*Significant with p < 0.05.

### Ultrasound imaging of core muscles activity

The VL group had significantly lower PFM displacement, diaphragmatic excursion, and lumbar multifidus thickness than the control group (p < 0.05). However, the ratio of transversus abdominis activation did not differ significantly between groups (p > 0.05) (Table 2).

**Table 2:** Ultrasound imaging of core muscle activity for both groups.

	VL group (n = 48)	Control group (n = 52)	MD (95% CI)	t-value	p-value
PFM displacement	0.29 ± 0.16	0.71 ± 0.23	− 0.42 (− 0.49: − 0.33)	− 10.08	0.001*
Diaphragmatic excursion	2.65 ± 0.56	5.40 ± 0.43	− 2.75 (− 2.95: − 2.55)	− 27.66	0.001*
Transversus abdominis activation ratio	1.65 ± 0.31	1.59 ± 0.26	0.06 (− 0.05: 0.17)	0.97	0.33
Lumbar multifidus thickness	20.94 ± 10.30	31.02 ± 11.11	− 10.08 (− 14.32: − 5.82)	− 4.70	0.02*

Values are expressed as mean ± standard deviation.

*PFM* pelvic floor muscle, *VL* vaginal laxity, *MD* mean difference, *CI* confidence interval, *p-value* probability.

*Significant with p < 0.05.

### Arabic female sexual function index (ArFSFI)

The VL group had significantly lower ArFSFI scores than the control group (p < 0.05) (Table 3).

**Table 3:** ArFSFI scores in both groups.

	VL group (n = 48)	Control group (n = 52)	MD (95% CI)	t-value	p-value
ArFSFI	19.96 ± 3.41	29.16 ± 3.61	− 9.2 (− 10.59: − 7.81)	− 13.11	0.001*

Values are expressed as mean ± standard deviation.

*ArFSFI* Arabic female sexual function index, *VL* vaginal laxity, *MD* mean difference, *CI* confidence interval, *p-value* probability.

*Significant with p < 0.05.

## Discussion

Vaginal laxity, an aspect of pelvic floor dysfunction that lacks comprehensive understanding^4^, is often addressed in physical therapy rehabilitation programs by primarily targeting the PFMs^9,10^, without incorporating training for other integral core muscle groups. Hence, this study aimed to assess the activity of core muscles through ultrasound imaging in multiparous women with VL compared to a control group without VL. By addressing the compromised core muscle activity associated with VL, comprehensive physical therapy rehabilitation programs can be developed to restore optimal core muscle function.

The study revealed that the group with VL had significantly lower PFM displacement, diaphragmatic excursion, lumbar multifidus thickness, and ArFSFI scores than the control group. However, the transversus abdominis activation ratio did not reveal a significant difference between both groups.

In terms of PFM displacement, assessed during maximal contraction, a noteworthy reduction was noted in the group experiencing VL compared to the control group. This result could be supported by Manzini et al.^6^, who demonstrated a notable increase in levator hiatal area during maximum Valsalva maneuver among women who reported VL, reinforcing the accumulating evidence linking VL to increased pelvic floor dispensability. They ascribed this abnormal enlargement in levator ani hiatal dimensions to injury of the levator ani muscle through macro-trauma (complete detachment) or micro-trauma (excessive stretching). Also, Moegni and Christian^37^ concluded that women with both pelvic organ prolapse and hiatal ballooning exhibited weaker levator ani muscles than those with no hiatal ballooning. Thus, we can say that the lower displacement of PFM reported in the group with VL in our study might be associated with the occurrence of hiatal ballooning frequently observed in cases of VL.

Regarding diaphragmatic excursion, assessed at the expiratory phase end, it was significantly decreased in the group with VL in comparison to the normal controls. This result could be related to the synergistic action between the diaphragm and the PFMs. During inspiration and expiration, PFMs contract and relax in synchrony with the diaphragm. Additionally, PFMs become more active when there is a rise in intra-abdominal pressure, as seen during forceful expiration or coughing. This suggests that the PFMs significantly contribute to the coordination between the diaphragm and abdominal muscles for maintaining appropriate intra-abdominal pressure^38^. Therefore, the reduced diaphragmatic excursion noted in the VL group in the current study might be associated with the lower PFM displacement reported in this group.

Concerning lumbar multifidus thickness, reported as a percent of change or ratio between rest and contraction thickness, a significant decrease was discovered in the VL group compared to the normal controls. The lumbar multifidus muscle is a key contributor to spine stability and is recognized as a fundamental element of the core musculature. Alongside the PFMs, diaphragm, and transversus abdominis, these muscles form a cohesive unit that creates a cylindrical structure. Working together, they generate lumbopelvic forces that enhance trunk stability, control, and effective movement. Additionally, this coordinated effort improves balance, coordination, postural stability, and alignment while also supporting continence^11,12^. Research conducted by Hides et al.^39^ revealed an improvement in the multifidus muscle’s cross-sectional area following a stabilization training program that targeted the PFMs in conjunction with training the multifidus and abdominal muscles in young elite cricketers experiencing low back pain, confirming the synergistic interaction among these muscles. Based on our findings, the decreased lumbar multifidus thickness seen in the VL participants could potentially be attributed to the comparatively lesser displacement exerted by the PFMs within this particular group.

Interestingly, the transversus abdominis activation ratio, obtained through the division of the thickness during ADIM by the thickness at rest, did not differ significantly between both groups in the current study despite the significant differences detected in the ultrasound imaging of other core muscle activity. This finding could be supported by de Abreu et al.^40^ who demonstrated that women experiencing low back pain exhibit normal activation of the transverse muscle, despite experiencing diminished force in PFM contractions.

The lack of significant difference in transversus abdominis activation ratio between the two groups in our study may initially appear surprising. However, it could be related to the broad anatomical attachment of the transversus abdominis muscle, which might offer protection against weakness in patients with VL. The transversus abdominis muscle wraps around the front and sides of the abdomen, creating a belt-like structure with its horizontal fibers. It is broad with attachments to the lumbar vertebrae via the thoracolumbar fascia, as well as to the pelvis and ribcage. It also traverses the sacroiliac joints and helps stabilize the sacrum between the hip bones^41^.

The reduced scores of ArFSFI observed in the VL group indicated that their sexual functioning was not as good as the normal control group. This result aligned with a previous study, which reported that VL exhibits a strong association with reduced vaginal sensation during coitus, as well as a deterioration in overall sexual health^3^. Additionally, Qureshi et al.^42^ found that out of the women diagnosed with VL, 46% experienced sexual distress based on the criteria of the female sexual distress scale-revised, while the female sexual dysfunction criteria were met by 65% of them according to the FSFI. Moreover, according to Pauls et al.^43^, a majority of urogynecologists (57%) expressed the belief that the presence of VL adversely impacts the quality of life, sexual functioning, and relationship satisfaction of their patients.

The reduced sexual function observed in the VL group might be attributed to reduced friction, feelings of looseness, and difficulty reaching orgasm during intercourse^44^. In addition, diminished vaginal sensation during sexual intercourse in VL women could be caused by anatomical injury to the perineal body, vaginal canal, or introitus, as well as nerve and connective tissue damage from pregnancy and normal vaginal delivery, or collective influence of these issues^45^. A recent research conducted by Sartori et al.^46^ revealed a connection between sexual function and the strength of PFMs. Therefore, the reduced PFM displacement seen in the VL group via ultrasound likely contributes to reduced sexual function. Trauma to the PFMs during gestation and vaginal birth is associated with an enlargement of the genital hiatus, which in turn adversely affects female sexual function in individuals with VL^9^. PFM malfunction can impede proper blood supply to the clitoris, reduce orgasmic capacity, and hinder sexual pleasure^47^. Furthermore, predicting changes in core muscles help clinicians to effectively deal women with sexual dysfunction^48^.

### Strengths and limitations

The present study exhibits multiple strengths. It is the first of its kind to compare core muscle activity using ultrasonography between multiparous women with and without VL. The ultrasound imaging was performed by a skilled and experienced physical therapist, providing a non-invasive and reliable method to measure core muscle activity. The study also employed a validated questionnaire to assess the sexual functioning of the participants. Furthermore, the sample size was calculated before the study to ensure adequate statistical power. Lastly, the inclusion of a control group of women without VL allowed for a comparison of sexual function and core muscle activity between the two groups.

There are specific limitations in the study that necessitate acknowledgment. Firstly, the cross-sectional design of the study prevents us from examining the long-term relationships between the factors being investigated and the outcomes. Additionally, the classification of participants into groups was based on their self-reported VL using the VLQ. It is noteworthy to mention that the VLQ lacks validation as a research tool. Nevertheless, a validated tool exclusively designed for evaluating VL is currently unavailable. As a result, the VLQ was employed as the sole choice for assessing VL in this context. Therefore, it’s essential to highlight the need for employing alternative assessment methods, such as measuring the hiatal area via ultrasound, to enhance the accuracy of VL evaluations. Moreover, manual vaginal palpation, which could provide further insights into VL, the strength of the vagina, and the PFMs, was not incorporated into the evaluation process. A suboptimal approach was used to evaluate PFM function, with transabdominal ultrasound chosen over much more precise alternatives such as transperineal ultrasound or a simpler, more accurate methods like digital palpation. Furthermore, the assessment was conducted by evaluating each muscle group independently and through different tasks, rather than evaluating their combined function as a cohesive group. This limitation should be explicitly acknowledged and taken into consideration when interpreting the findings and drawing conclusions.

## Conclusion

Multiparous women with VL experienced reduced movement in their pelvic floor muscles, limited diaphragmatic excursion, and a thinner lumbar multifidus muscle when compared to the control group. However, there was a non-significant difference in the transversus abdominis activation ratio between the two groups. These findings emphasize the importance of physical therapy interventions in managing VL. Physical therapy can address pelvic floor malfunction, diaphragmatic dysfunction, and lumbar muscle integrity through targeted exercises and therapeutic techniques. Focusing on these specific concerns in women with VL, physical therapy holds the promise of strengthening the pelvic floor, restoring diaphragmic mobility, and fostering overall core stability.

## Data Availability

Data will be held with the research author and maybe available upon request from corresponding author (Doaa A. Abdel Hady).
